# Building local human resources to implement SLMTA with limited donor funding: The Ghana experience

**DOI:** 10.4102/ajlm.v3i2.214

**Published:** 2014-11-03

**Authors:** Bernard Nkrumah, Beatrice van der Puije, Veronica Bekoe, Rowland Adukpo, Nii A. Kotey, Katy Yao, Peter N. Fonjungo, Elizabeth T. Luman, Samuel Duh, Patrick A. Njukeng, Nii A. Addo, Fazle N. Khan, Celia J.I. Woodfill

**Affiliations:** 1 US Centers for Disease Control and Prevention, US Embassy, Ghana; 2 Global Health Systems Solutions, C75/20 Amanfro Street, Abelenkpe, Ghana; 3 National AIDS Control Program, Ghana Health Service, Ghana; 4 National Public Health Reference Laboratory, Ghana Health Service, Ghana; 5 US Centers for Disease Control and Prevention, Atlanta, United States; 6 Global Health Systems Solutions, Cameroon; 7 US Centers for Disease Control and Prevention, Cote d’Ivoire

## Abstract

**Background:**

In 2009, Ghana adopted the Strengthening Laboratory Management Toward Accreditation (SLMTA) programme in order to improve laboratory quality. The programme was implemented successfully with limited donor funding and local human resources.

**Objectives:**

To demonstrate how Ghana, which received very limited PEPFAR funding, was able to achieve marked quality improvement using local human resources.

**Method:**

Local partners led the SLMTA implementation and local mentors were embedded in each laboratory. An in-country training-of-trainers workshop was conducted in order to increase the pool of local SLMTA implementers. Three laboratory cohorts were enrolled in SLMTA in 2011, 2012 and 2013. Participants from each cohort attended in a series of three workshops interspersed with improvement projects and mentorship. Supplemental training on internal audit was provided. Baseline, exit and follow-up audits were conducted using the Stepwise Laboratory Quality Improvement Process Towards Accreditation (SLIPTA) checklist. In November 2013, four laboratories underwent official SLIPTA audits by the African Society for Laboratory Medicine (ASLM).

**Results:**

The local SLMTA team successfully implemented three cohorts of SLMTA in 15 laboratories. Seven out of the nine laboratories that underwent follow-up audits have reached at least one star. Three out of the four laboratories that underwent official ASLM audits were awarded four stars. Patient satisfaction increased from 25% to 70% and sample rejection rates decreased from 32% to 10%. On average, $40 000 was spent per laboratory to cover mentors’ salaries, SLMTA training and improvement project support.

**Conclusion:**

Building in-country capacity through local partners is a sustainable model for improving service quality in resource-constrained countries such as Ghana. Such models promote country ownership, capacity building and the use of local human resources for the expansion of SLMTA.

## Introduction

The recent drive by the World Health Organization’s Regional Office for Africa (WHO AFRO) and the US President’s Emergency Plan for AIDS Relief (PEPFAR) toward strengthening laboratory systems in Africa is a historic step in the improvement of health systems. However, this effort is hampered by the lack of locally-qualified laboratory personnel.^1,2^ The need to strengthen weak laboratory networks, systems and services in developing countries was highlighted in 2008 in a series of advocacy meetings: the Maputo Declaration (January 2008) for strengthening laboratory health systems,^1^ the Lyon Statement (April 2008) on the need for developing countries to establish practical quality management systems,^3^ and the Yaoundé Resolution (September 2008) issued by WHO AFRO in recognition of the dilapidated state of the laboratory health systems and the need to strengthen all laboratory tiers in order to fight multiple diseases.^4^ In the following year in Kigali (July 2009), WHO AFRO launched a stepwise laboratory accreditation preparation scheme, which recognises and encourages incremental progress toward fulfilment of the requirements of the International Organization for Standardisation (ISO) 15189 standard; and, at the 59th session of the WHO AFRO Regional Committee (September 2009), Member States adopted resolutions AFR/RC59/R2 and AFR/RC59/WP/3 aimed at strengthening public health laboratories and other centres of excellence in order to improve disease prevention and control.^4^ Also launched in Kigali was the Strengthening Laboratory Management Toward Accreditation (SLMTA) programme, developed by the US Centers for Disease Control and Prevention (CDC) and partners so as to guide countries toward achieving the called-for improvements. As PEPFAR’s flagship programme for strengthening laboratory systems, SLMTA has been implemented in 47 countries, demonstrating measurable and positive impact.^5^

Ghana has more than 420 public sector laboratories organised into a four-tier laboratory system: national/central, regional/zonal, district and subdistrict levels. National- and regional-level laboratories provide technical assistance and supportive supervision for the district and subdistrict level laboratories. In 2012, the Ministry of Health (MoH) and the Ghana Health Service (GHS) drafted a five-year national laboratory strategic plan. This strategic plan clearly addressed local capacity building and highlighted the government’s commitment to fulfil their mandate of providing quality healthcare through the adoption of SLMTA as the systematic approach for implementation of laboratory quality management systems (QMS) in Ghana. In conjunction with the strategic plan, Ghana drafted a national laboratory accreditation policy. Accreditation ensures the quality, precision, dependability and timeliness of laboratory testing results.^6,7,8^ Two private laboratories in Ghana are accredited, but none of the public sector laboratories, which perform the bulk of patient testing, are accredited to any national or international standards.

Called the largest health initiative ever implemented by a single country to address a disease, since 2003 PEPFAR has provided financial and technical support to developing countries to fight HIV, saving millions of lives.^9^ Laboratory strengthening is a critical component of the PEPFAR strategy. In 2013, the Institute of Medicine report on PEPFAR noted that ‘its substantial support for laboratory strengthening has had fundamentally positive effects for the response to HIV and has been leveraged to improve the functioning of entire health systems’.^9^ Ghana is one of PEPFAR’s Targeted Assistance countries, which receive limited financial support for key populations or priority technical areas, capacity building and/or technical assistance.^10^ Ghana’s annual laboratory budget from PEPFAR is about $1.1M (8% of the total Ghana PEPFAR funding). This notwithstanding, PEPFAR is a major source of support for Ghana’s laboratory system strengthening programmes. Given the limited funding, Ghana sought a strategy to implement the SLMTA programme for laboratory system strengthening in an economical and sustainable manner. This paper describes how a country like Ghana, which receives very limited funding, was able to achieve marked improvement in laboratory quality management by empowering local partners to implement the SLMTA programme.

## Research method and design

### Programme implementation approach

A top-down programme implementation approach was adopted. Under this model, the country sought to first build capacity and strengthen the quality of laboratory services within the national- and regional- level laboratories. In turn, these higher-level laboratories would be equipped to support capacity building and strengthen quality laboratory services at the lower tier levels. Two local implementing partners, the governmental agency GHS and a non-government not-for-profit organisation, Global Health Systems Solutions (GHSS), were engaged by the CDC’s Ghana office to implement SLMTA through cooperative agreements.

### Baseline audits and site selection

Eighteen public sector laboratories (three national, 12 regional and three district level) were considered initially for enrolment into the SLMTA programme. GHS conducted a baseline audit of all 18 laboratories using the Stepwise Laboratory Quality Improvement Process Towards Accreditation (SLIPTA) checklist, which provides a quantitative measure of adherence to ISO 15189 requirements. The scored checklist quantifies a laboratory’s quality status using a zero- to five-star rating: 0–141 points (< 55%) = zero stars, 142–166 points (55% – 64%) = one star, 167–192 points (65% – 74%) = two stars, 193–218 points (75% – 84%) = three stars, 219–243 points (85% – 94%) = four stars and 244–258 points (95% – 100%) = five stars.^11^

**FIGURE 1:**
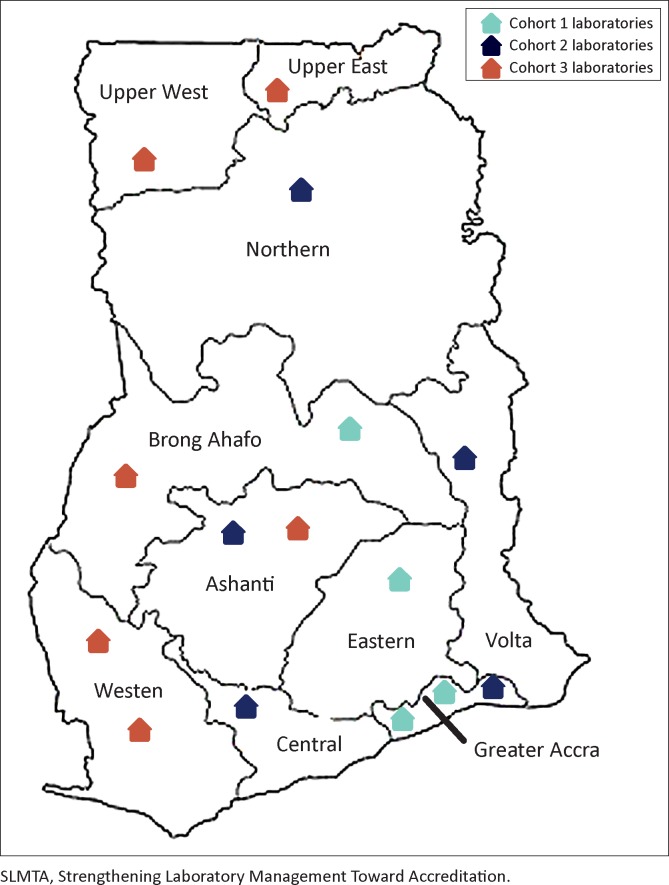
Geographic location of laboratories involved in SLMTA implementation in Ghana, 2011–2013.

Fifteen of the 18 audited laboratories were selected to enrol in the SLMTA programme based on several factors: their baseline scores; infrastructural availability and suitability; geographical distribution; staffing; and management and staff willingness to participate. The 15 laboratories comprised two national-level laboratories, 12 regional-level laboratories and one district-level laboratory (Table 1). Geographically, these laboratories cover all 10 regions in Ghana (Figure 1). These laboratories were grouped further into three cohorts: Cohort 1 comprised four laboratories; Cohort 2, five laboratories; and Cohort 3, six laboratories.

**TABLE 1:** Level and function of laboratories in the SLMTA programme.

Laboratory Code	Tier	Function
L1	Regional	Diagnostic
L2	Regional	Diagnostic
L3	National	Teaching hospital
L4	National	Public health
L5	Regional	Diagnostic
L6	Regional	Diagnostic
L7	Regional	Public health
L8	Regional	Diagnostic
L9	Regional	Diagnostic
L10	Regional	Diagnostic
L11	Regional	Public health
L12	Regional	Diagnostic
L13	Regional	Public health
L14	Regional	Diagnostic
L15	District	Research

SLMTA, Strengthening Laboratory Management Toward Accreditation.

### Development of a local mentorship programme

In October 2011, GHSS, working with GHS and other stakeholders, initiated a local mentorship programme, training and placing full-time local mentors at each of the selected laboratories. Potential mentors were recruited through newspaper advertisements in an open competitive process; minimum qualifications included a degree in Medical Laboratory Technology or equivalent and previous experience in working with GHS. Their training included classroom-based lectures on topics such as QMS and the 12 Quality System Essentials (QSEs); ISO 15189 requirements; conducting audits using the SLIPTA checklist; and project management. Trainee mentors then spent one to three weeks with GHSS senior mentors during a field practicum before they were sent to their post. Mentors were closely supervised by GHSS, GHS and CDC by means of a weekly and monthly reporting systems.

### SLMTA programme implementation

The first cohort of laboratories implemented SLMTA from April 2011 to April 2012, the second from May 2012 to May 2013 and the third from February 2013 to November 2013. Four staff members from each enrolled laboratory, namely, the laboratory manager, quality manager and two other staff members, participated in the three SLMTA workshops which were coordinated by GHSS and GHS. Laboratories selected their own participants, with assistance from their upper management, by following the above-mentioned selection criteria. SLMTA implementation in the three cohorts followed the prescribed SLMTA process,^12^ with the three workshops taught by qualified SLMTA facilitators from GHS and GHSS, implementation of improvement projects after each workshop and quarterly site visits to monitor progress, track quality indicators and provide technical assistance. Participants also attended complementary training on ISO 15189 and internal audit conducted by the Ghana Standards Authority (GSA). Site visits were conducted using the SLMTA site visit monitoring tool by either GHSS alone or by a combined technical team from GHS, CDC’s Ghana Office and GHSS. Written reports detailing observations, technical assistance provided and recommendations were delivered to the laboratory, the Regional Directors of Health and Hospital Directors in order to ensure that management within each region were well informed regarding the programme and the progress made by their laboratories.

### Programme monitoring and evaluation

Intermediate audits were conducted semi-annually in order to measure the progress made by the laboratories whilst helping partners to review their work plan, implementation approach and the mentorship programme. The audits were conducted by trained in-country auditors using the SLIPTA checklist and results were communicated to the participating laboratories so as to guide corrective actions. Mentors who were cross-trained as auditors did not conduct audits in laboratories that they mentored. An exit audit was conducted for each laboratory in the three cohorts at the end of the SLMTA training. Follow-up audits were conducted six months after the exit audits in order to monitor the performance of the laboratories and to ensure that recommendations from the exit audits were addressed. One laboratory from Cohort 3 (L12) did not receive an intermediate audit because of delayed communication to the site. Another laboratory in Cohort 3 (L15) did not receive an exit audit because of its high scores at baseline and intermediate audits; in November 2013, this laboratory and the 3 highest-performing laboratories from Cohort 1 underwent official SLIPTA audits by the African Society for Laboratory Medicine (ASLM).

Additional indicators, such as specimen rejection rates and patient satisfaction, were also tracked in Cohort 1 laboratories so as to assess the progress and impact of implementation. Specimen rejection rates were calculated as a percentage of samples rejected as a result of non-conformity to specimen acceptance criteria. Because of a lack of data on the specimen rejection rate in Ghanaian laboratories prior to the implementation of SLMTA, specimen rejection rates were only monitored after baseline, during SLMTA implementation and thereafter for three years. Patient satisfaction was measured using questionnaires given to patients and comments received from patients through the suggestion box.

The cost in US dollars for the implementation of SLMTA was reported by the implementing partners. Estimated costs included mentor salaries, SLMTA training and improvement project support. Salaries of mentors were determined by the implementing partners, in accordance with Ghana’s labour laws. SLMTA training costs were calculated based on four personnel from each laboratory and two trainers participating in the three five-day workshops. Costs included per diem, local transportation to the training venue, training materials for all participants and the workshop venue package. Workshop trainers were staff members from the implementing partner; their salaries were considered to be an in-kind contribution and were not included in the estimates. Similarly, salary and time missed from work for participants were not included. Expenditures sustained by the partner in order to support laboratory improvement projects, such as colour-coded bin liners, emergency eye-wash kits and ISO training for internal auditors, were also estimated.

### Preparation for SLMTA expansion

In April 2013, a laboratory auditors training course was organised by the Clinical and Laboratory Standards Institute (CLSI) and ASLM for selected professionals with experience in QMS, ISO 15189 and SLMTA. The training programme equipped the participants to plan, prepare and conduct independent laboratory quality audits based on the SLIPTA checklist and ISO 15189 requirements. The training format consisted of classroom didactic presentations and a field practicum through mock audits.

In September 2013, a SLMTA training-of-trainers (ToT) workshop was conducted in order to increase the pool of local trainers and implementers for nationwide SLMTA scale-up. This workshop was led by two SLMTA master trainers from Ghana and one from Nigeria, all previously trained to lead ToT workshops.^13^ Local laboratory professionals who had implemented SLMTA were selected for the training.

### Data entry and analysis

The results of the audits were entered into a Microsoft® Excel spreadsheet (Microsoft® 2010). Statistical analysis was done using Stata SE 12.1 (Stanford University IT Services 2012) after the data had been cleaned and exported. Continuous variables such as SLIPTA scores were summarised and presented as medians. Percentage scores were determined by dividing the respective scores by the maximum possible points and expressing the results as a percentage.

## Results

### Measuring the impact of SLMTA

**FIGURE 2:**
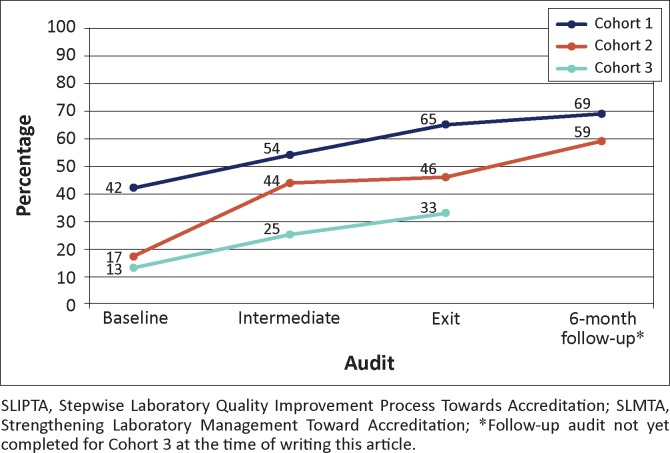
Median SLIPTA scores for the three Ghana SLMTA cohorts.

**FIGURE 3:**
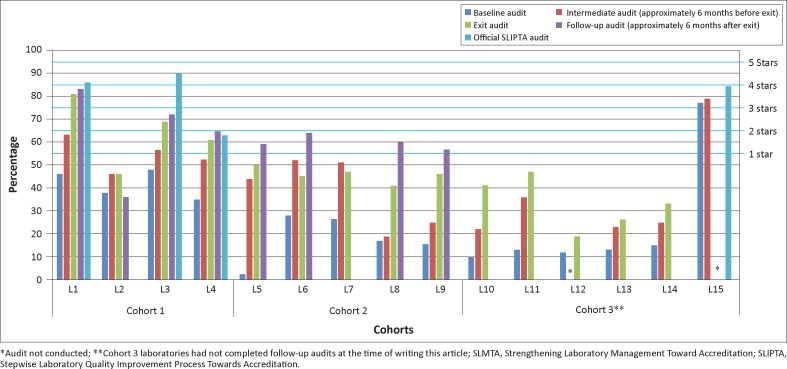
Performance of 15 Ghana SLMTA laboratories at baseline, intermediate, exit, follow-up and official SLIPTA audit.

Laboratories in all three cohorts demonstrated a steady improvement in the implementation of QMS, as was illustrated by the median SLIPTA scores at each audit (Figure 2). Median improvements from baseline to exit were 23 percentage points for Cohort 1, 29 percentage points for Cohort 2 and 20 percentage points for Cohort 3. The most improved laboratory (L5) demonstrated an increase from 2% at baseline to 50% at exit, which increased further to 59% at the follow-up audit six months later. Laboratory 2, on the other hand, scored 38% at baseline and 46% at exit, but decreased to 36% at the follow-up audit (Figure 3). At baseline, only one of the 15 laboratories was at the one-star level. By the exit audit, three laboratories had reached at least one star; of the eight laboratories that conducted follow-up audits, seven had reached at least one star.

**FIGURE 4:**
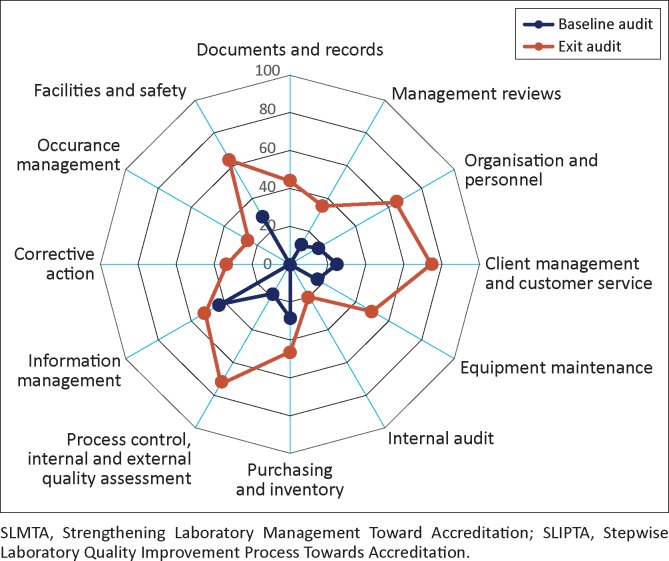
Median performance of all 15 Ghana SLMTA laboratories across the 12 Quality Systems Essentials, as measured by the SLIPTA checklist at the baseline and exit audits.

Each of the 12 QSEs improved from baseline to exit. The most improved areas were process control and internal and external quality assessment (54%); customer service (50%); organisation and personnel (48%); and documents and records (44%) (Figure 4). Information management showed the least improvement (9%), followed by purchasing and inventory (18%) and internal audit (20%). The areas with the lowest median exit audit scores were internal audit (20%), occurrence management (25%), corrective action (33%) and management reviews (35%).

The three highest-performing laboratories at exit audit (L1, L3 and L4, all from Cohort 1), plus L15 from Cohort 3, which did not receive an exit audit but earned three stars at both the baseline and intermediate audits, underwent official SLIPTA audits by ASLM. Official SLIPTA audit results overall were slightly higher than exit audit results (Figure 3). Three of these laboratories (L1, L3 and L15) earned four official SLIPTA stars and one (L4) earned one star.

**FIGURE 5:**
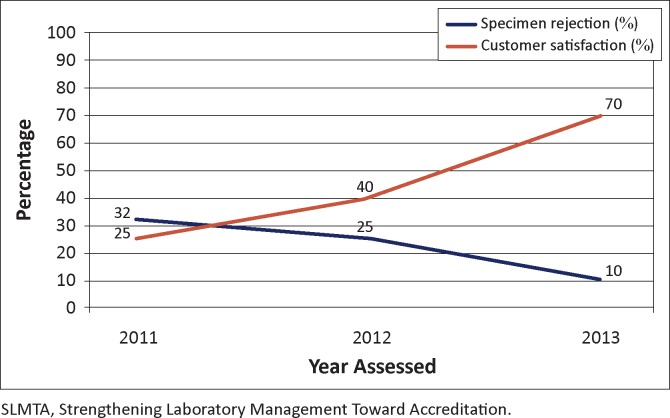
Specimen rejection and customer satisfaction trends for SLMTA Cohort 1 laboratories.

Average specimen rejection rates across the four SLMTA laboratories in Cohort 1 decreased from 32% (range 23% – 44%) in 2011 to 25% (range 12% – 35%) in 2012 and 10% (range 3%–12%) in 2013. Patient satisfaction increased from 25% to 70% over the same time period (Figure 5).

### Local capacity building and cost of SLMTA implementation

The Ghana SLMTA team presently comprises 18 SLMTA trainers, 15 mentors, 11 CLSI/ASLM trained auditors and two master trainers. This team has been responsible for the successful implementation of three cohorts of SLMTA with a total of 15 laboratories, training 60 laboratory professionals in QMS. To date, two senior mentors and six trainers have completed phase one of the SLIPTA auditor training conducted by ASLM and CLSI.

The cost for SLMTA implementation for each laboratory was incurred in the areas of project management, SLMTA trainings and embedded mentorship. Taken together, the implementing partners reported a total expenditure of $600 000 to implement SLMTA in the 15 laboratories. On average, $40 000 total was spent per laboratory to cover mentors’ salaries ($24 000), SLMTA training ($6000) and improvement project support ($10 000).

## Discussion

The Ghana National Laboratory Strategic Plan prioritises the development of local capacity as a sustainable way to support the delivery of quality results for improved patient care and treatment. In 2009, Ghana adopted SLMTA using limited funding and only local human resources to drive the programme forward. Results have been remarkable in the 15 laboratories enrolled in SLMTA – audit scores doubled from baseline to exit and more than half of those laboratories reached one or more stars, including three laboratories that have achieved four stars. The success of the Ghana SLMTA programme can be attributed to three strong factors: management engagement and commitment, the use of local partners and implementation of a local mentorship programme.

Management engagement and commitment have been shown to be critical elements in promoting quality laboratory services.^14^ In Ghana, management was engaged at three levels: central, regional and facility. In June 2012, shortly after completion of the first SLMTA cohort, GHS organised a one-day meeting, chaired by the Director General of GHS, to sensitise regional health directors, hospital medical directors and laboratory managers from all 10 regions of the country on the SLMTA programme and how it could benefit their facilities. A medical director whose facility had recently completed SLMTA gave a presentation on how the programme had transformed his facility. This medical director became the ‘SLMTA ambassador’ amongst his peers. The meeting served as a catalyst in the acceleration of SLMTA implementation in Ghana; afterward, regional directors of health engaged regularly with participating laboratories by means of site visits, courtesy calls and review of audit reports. Some medical directors at the facility level even joined in-house SLMTA trainings, site visits and debriefing activities. Others personally took on responsibilities to oversee some improvement projects. This high-level engagement created tremendous enthusiasm within the facilities and contributed to staff morale.

With a limited budget, it was important for Ghana to incorporate cost-effective and results-oriented approaches into its programme implementation. To achieve the country’s priority of developing sustainable human capacity, local partners were engaged from the beginning, which proved to be advantageous in several respects: (1) they were familiar with the local administration, culture and language; (2) they were well accepted into the facilities as peers; and (3) their service was less expensive than international partners. Local partners contributed directly to workforce development through the training and hiring of local human resources. Care was taken to ensure that hiring was done through a merit-based process and was well specified in the organisations’ policies and procedures. Key components included training, capacity building, salary structure and working conditions for technical, administrative and financial staff. To ensure that programme objectives and targets were met, partners were assisted in adhering to reporting requirements in a timely and consistent manner.

Mentorship is an important vehicle to establish and solidify QMS and to help laboratories achieve their quality improvement goals.^15^ Guidance regarding the implementation of a structured laboratory mentorship programme has been documented.^15^ Similar to the Lesotho mentorship approaches,^16,17^ Ghana adopted a full-time, resident mentorship approach. In this approach, each mentor was assigned to a laboratory and resided within the locality where the laboratory was situated. Effective mentoring requires full understanding of a laboratory’s culture, processes, procedures and people; we found that this embedded mentorship approach was further enhanced with the placement of indigenous professionals who already understood the in-country laboratory culture. It has been suggested that mentorship visits of four to eight weeks may be more effective than shorter periods.^17^ In our embedded mentorship approach, mentors stayed at the facility full time for the duration of the programme (18–24 months) and worked with laboratory staff to help raise the laboratory’s level of performance.

One positive result of SLMTA implementation in Ghana was improved patient satisfaction in the quality of service delivery and a reduction in the rejection of specimens. These results are consistent with previously-published findings.^7^ Patient satisfaction was improved by the introduction of: customer service managers; suggestion boxes; client satisfaction surveys; and posters which displayed in bold text the cost of tests, expected turnaround time for tests and other vital information to patients. Specimen rejection was reduced through three interventions: development of quality manuals that defined clearly the policies, processes and procedures for all the laboratories; development of a clinicians’ handbook that defined in detail to all clinicians, nurses and midwives the specimen acceptance and rejection criteria for the laboratory; and consistent training for both laboratory and non-laboratory staff.

Despite the improvements, challenges remain in the SLMTA laboratories. Some QSEs, such as internal audit, occurrence management, corrective actions and management reviews, scored a median of ≤ 35% at the exit audit, indicating that these critical areas are not functioning adequately. These QSEs are common low-scoring areas, as reported in Lesotho^16^ and other countries.^18^ In Ghana, these deficiencies were largely a result of a lack of internal audit skills, inadequate staffing, limited experience of mentors, inefficient communication channels and institutional bottlenecks, such as administrative and procurement^7^ processes. As a result of these findings, training on internal audit has been conducted for all laboratory and quality managers, in collaboration with GSA. Inadequate staffing levels and lack of motivation amongst some staff members, coupled with increased workloads, may have contributed to delays in the implementation of improvement projects. These issues are not easy to address, although advocacy continues for the prioritisation of laboratory human resource needs and identification of incentive options for overworked staff. Many of the mentors used in these three cohorts had been trained recently and this was their first mentoring experience; whilst some settled in quickly, others needed more time to adjust to their new environment and tasks assigned. With time, these mentors have gained tremendous experience and are more effective in assisting laboratories to implement quality systems. Once SLMTA roll-out is complete, these mentors will have skills that will make them valuable assets as quality managers. Finally, inefficient communication and institutional bottlenecks remain a challenge; however at some facilities, especially in those where management was engaged in the SLMTA process, efforts are being made to simplify administrative processes and streamline communication channels.

Most countries implementing SLMTA have relied heavily on PEPFAR funding for implementation support. Limited PEPFAR funding in Ghana meant building local capacity that could be sustained and replicated as the SLMTA program grows. Because SLMTA activities such as training, mentoring, monitoring and auditing are inter-related, we adopted a cross-training approach such that SLMTA trainers were also trained as mentors and certified as auditors, in an effort to maximise their potential.

### Limitations of the study

One limitation of this study was the absence of control laboratories that were followed over the same period of time. As a result we could not compare the improvements with laboratories that were not enrolled in SLMTA. Whilst it is possible that some improvements observed in this programme were a result of secular influences or random factors, the magnitude of the observed impact strongly suggests a positive impact of the SLMTA programme.

### Recommendations

Local partners may be considered for in-country programme implementation. However, capacity building of partner staff in administrative, technical and financial areas must be an integral part of the programme to ensure utmost compliance with reporting requirements.

### Conclusion

The SLMTA programme in Ghana has shown substantial laboratory improvements as evidenced by progress in 15 laboratories, including four that have been audited officially by ASLM. This experience demonstrates that local partners, when supported and managed adequately, can achieve great results at a reasonable cost. Our programme also demonstrates the feasibility of indigenous capacity building and sustainability in an era of reduced PEPFAR funding, as countries are encouraged to do more with less.
